# Natural Nrf2 activators modulate antioxidant gene expression and apoptosis in leukemic K-562 cells

**DOI:** 10.1007/s12032-025-02946-4

**Published:** 2025-07-31

**Authors:** Iason-Spyridon Patergiannakis, Sofia K. Georgiou-Siafis, Lefkothea C. Papadopoulou, Ioannis S. Pappas

**Affiliations:** 1 Laboratory of Veterinary Pharmacology and Toxicology, Faculty of Veterinary Medicine, University of Thessaly, Trikalon 224, 43100 Karditsa, Thessaly Greece; 2 Laboratory of Pharmacology, School of Pharmacy, Faculty of Health Sciences, Aristotle University of Thessaloniki, 54124 Thessaloniki, Macedonia Greece

**Keywords:** Natural products, Nrf2, K-562 cells, Redox signaling, Apoptosis, *HO-1*

## Abstract

Natural products (NPs) have long been used in traditional medicine and continue to be explored for their chemopreventive and therapeutic properties. Many of their biological effects are mediated through redox-sensitive pathways, such as the nuclear factor erythroid 2-related factor 2 (Nrf2), a key regulator of antioxidant responses and cellular defense mechanisms. This study aimed to evaluate the antioxidant capacity, cytotoxicity, pro-apoptotic activity, and Nrf2-modulating effects of 22 natural product extracts traditionally used in ethnomedicine in a human leukemic K-562 cell model. The extracts were assessed for total phenolic content (TPC), radical scavenging activity (DDPH and ABTS assays), cytotoxic effects (AlamarBlue assay), apoptosis induction (caspase-3 activity), and regulation of Nrf2 target genes (*HO-1, NQO1* and *GCLC)* via RT‒qPCR. The extracts exhibited a wide range of antioxidant and cytotoxic activities. *Punica granatum, Rhodiola rosea,* and *Thymus vulgaris* showed high phenolic content and antioxidant potential. *R. rosea* displayed the most potent cytotoxic effect, whereas *Hypericum perforatum* induced the highest caspase-3 activity. Gene expression revealed that *T.vulgaris, R.rosea, Moringa oleifera, Withania somnifera, Saliva officinalis* and *Euphrasia officinalis* upregulated all three Nrf2 target genes, with *T.vulgaris* showing the greatest induction. These findings highlight the pharmacological potential of traditionally used natural products as modulators of redox signaling and apoptosis inducers in leukemia cells. The activation of the Nrf2 pathway by the selected extracts supports further investigation of their potential.

**Supplementary Information:**

The online version contains supplementary material available at 10.1007/s12032-025-02946-4.

## Introduction

Natural products (NPs) correspond to a wide variety of molecules with different structures, along with their evolutionary optimization to interact with proteins of other organisms, increasing their chemopreventive potential [1]. Moreover, plant products are often more accessible, chemically diverse, and better tolerated by patients. They are also associated with fewer adverse effects compared to synthetic compounds, making them valuable tools for research and drug development [2]. A variety of plant products have been used at times for therapeutic purposes, as they have diverse biological activities [1]. Numerous NPs have been traditionally used for therapeutic purposes across various cultures and continue to be studied for their diverse biological activities.

*Boswellia spp.*, including *Boswellia sacra* (frankincense), have been traditionally employed for inflammatory conditions and are also used as supportive therapies in cancer patients [3]. *Calendula officinalis* (pot marigold) has a long-standing use in wound healing and, more recently, has attracted attention for its anti-carcinogenic potential [4]. In addition to its ophthalmic applications, *Euphrasia officinalis* (eyebright) exhibits antioxidant and anti-proliferative activities [5]. *Fagonia cretica* (virgin’s mantle), commonly consumed as tea, has demonstrated anti-inflammatory and cytotoxic properties [6]. *Hypericum perforatum* (St. John’s wort) has been used traditionally for a wide spectrum of ailments and exhibits antitumor and analgesic activity [7]. *Matricaria chamomilla* (chamomile) has been traditionally used in medicine for its calming effects, and it exhibits a variety of biological properties, including hypotensive, hypoglycemic, antioxidant and anticancer activities [8].

Several dietary plants also demonstrate promising pharmacological properties. *Moringa oleifera* (drumstick tree)*,* known for its nutritional value, displays anticancer and antidepressant activities [9]. *Morus alba* (white mulberry)*, Ocimum basilicum* (basil)*, Olea europaea* (common olive) and *Origanum vulgare* (oregano) are traditionally used as food or herbal remedies and are now investigated for their cytotoxic, antioxidant, antimicrobial, and immunomodulatory properties [10–14]. Propolis, a complex bee product historically used in the mummification process, has documented antimicrobial and antitumor properties [15].

Other NPs commonly used in ethnomedicine, such as *Punica granatum* (pomegranate)*, Rhodiola rosea* (roseroot)*, Ribes nigrum* (black currant)*, Rosa canina* (rosehip), and *Rosmarinus officinalis* (rosemary), are used in ethnomedicine for gastrointestinal, respiratory, and stress-related conditions. These plants exhibit strong antioxidant and cytotoxic activities in vitro [16–21]. *Salvia officinalis* (sage)*, Sideritis scardica* (ironwort)*,* and *Silybum marianum* (milk thistle) are used in folk medicine for various treatments and show promising effects on redox regulation and cancer-related medicine [22–24].

Finally, *Thymus vulgaris* (thyme) and *Withania somnifera* (ashwagandha) are extensively studied species, with reported effects on inflammation, stress, sedation, and cancer, among other conditions [25, 26]. Many plant extracts and phytochemicals exert their antioxidant, cytoprotective, and anticancer effects, often through redox-sensitive pathways such as Nrf2. Several phytochemicals are Nrf2 activators [27, 28]. Plants produce many bioactive compounds in the form of secondary metabolites that have highly variable properties, including but not limited to, antitumor, antioxidant, and anti-inflammatory properties [29]. Phytochemicals target the Nrf2 pathway as they act through interaction with Keap1, disruption of the Nrf2‒Keap1 complex, epigenetic modifications and regulation of other molecules affecting the signaling pathway [29].

Nuclear factor erythroid 2-related factor 2 (Nrf2), encoded by the *NFE2L2* gene, is a key transcription factor that orchestrates the cellular response to oxidative stress. It binds to DNA via antioxidant response elements (AREs), and its action is hindered by Kelch-like ECH-associated protein 1 (Keap1) [30]. Under basal conditions, Nrf2 is sequestered in the cytoplasm by Keap1, which promotes its ubiquitination and subsequent degradation [31]. Upon oxidative or electrophilic stress this repression is alleviated, allowing Nrf2 to translocate into the nucleus and activate gene transcription by binding to AREs [31]. These factors are crucial for the regulation of the antioxidant response of the cell [32], and the activation of the Keap1-Nrf2 pathway protects the cell against inflammation and restores protein homeostasis [33]. As a result, this pathway has been targeted for the treatment of diseases such as multiple sclerosis [33]. Beyond redox regulation, Nrf2 is also involved in controlling cell survival, growth and proliferation [34]. It regulates the expression of numerous cytoprotective genes, including those encoding enzymes involved in drug metabolism and GSH synthesis, such as NAD(P)H quinone oxidoreductase 1 (*NQO1*), heme oxygenase 1 (*HO-1*) and the glutamate-cysteine ligase catalytic subunit (*GCLC*) [31, 35]. Moreover, Nrf2 plays a role in the regulation of cellular metabolism and is involved in the metabolic reprogramming of cancer [31]. Although Nrf2 has protective, anti-inflammatory, and anti-carcinogenic functions, it may also enhance drug excretion and support chemoresistance in cancer [35]. Consequently, both Nrf2 activators and inhibitors can be used against cancer, the first for the prevention of carcinogenesis and the second for cancer treatment [34].

In chronic myeloid leukemia (CML), Nrf2 appears to promote drug resistance and reduce apoptotic susceptibility in K-562 cells, a human erythroleukemia cell line frequently used in redox and cancer research [35]. Nevertheless, Nrf2 inducers may be used for the protection of normal cells against carcinogens and during cancer treatment, as cells within the tumor microenvironment and immune system are more susceptible to anticancer drugs because they express Nrf2 at regular levels [36]. Additionally, Nrf2 may contribute to the protection of red blood cells against heme toxicity during hemolytic events, as *HO-1* plays a pivotal role in the defense against hemin-induced cytotoxicity. Hemin orchestrates a Nrf2-centered defensive pathway by inhibiting the Keap1-dependent ubiquitination and degradation of Nrf2 in K562 cells [37]. Recently, GSH, as one of the key metabolites increased by free heme toxicity, was found to form adducts with hemin, leading to its chemical disintegration [38]. Therefore, Nrf2 activators might be used for the treatment of hemolytic disorders, as shown in K-562 pro-erythroid cells and other cell lines.

In this study, we evaluated 22 natural product extracts for their antioxidant capacity, pro-apoptotic effects, and regulatory impact on Nrf2-responsive genes in K-562 cells. A combination of radical scavenging assays, cytotoxicity and caspase-3 activity analyses, and RT-qPCR profiling of *HO-1*, *NQO1* and *GCLC* expression were used. We identified several extracts with potent redox-modulating and anti-leukemic properties.

## Materials and methods

### Chemicals, reagents and cell lines

All plant materials were purchased commercially from local producers and markets in Thessaly and Crete, Greece. ABTS was purchased from Fluorochem Ltd. (Glossop, UK). DPPH, dithiothreitol (DTT), FBS, Folin-Ciocalteu reagent, gallic acid, glycerol, guanidinium thiocyanate, phenol, phosphoric acid, sodium acetate, sodium carbonate, sodium chloride, and Trolox were purchased from Sigma‒Aldrich (St. Louis, MO, USA). The following materials were purchased from Thermo Fisher Scientific (Waltham, MA, USA): ammonium thiocyanate, Coomassie Blue G250, disodium phosphate, EDTA, ethylene glycol, ethanol (EtOH), isopropanol, Nonidet P40, potassium chloride, potassium phosphate, and potassium persulfate. Thermo Fisher Scientific, Waltham, Gibco (MA, USA), provided penicillin‒streptomycin. Apopain was purchased from Eurogentec (Seraing, Belgium). Chloroform was purchased from VWR International, LLC. (Radnor, Pennsylvania, USA). DMSO was purchased from PanReac AppliChem, Barcelona, Spain. The iTaq Universal One-Step RT‒qPCR Kit was purchased from Bio-Rad Laboratories (USA). RPMI-1640 was obtained from Biosera (France). K-562 cells were kindly donated by the Laboratory of Pharmacology, School of Pharmacy, Aristotle University of Thessaloniki, Thessaloniki, Greece. Bovine albumin serum was kindly donated by the Biochemistry Laboratory of the Faculty of Veterinary Science, University of Thessaly, Karditsa, Greece.

### Preparation of the plant extracts

The relevant parts of each plant were first pulverized into a fine powder. Different extraction solvents and times were used for each plant. The solvents used were ethanol, methanol and mixtures of ethanol and methanol with water, as they have different abilities to extract and isolate antioxidant secondary metabolites. The extraction was performed at room temperature. The samples were first soaked with the corresponding solvent and then filtered, concentrated in a rotary evaporator and resuspended in ethanol.

The differences in solvent systems and extraction times across plant species were based on established literature and aimed to optimize the recovery of diverse phytochemical classes. Solvent polarity was adjusted to match the known solubility of bioactive compounds in each plant, while extraction duration was tailored according to tissue texture and powder fineness. This rationale reflects common phytochemical practice, ensuring that each extract would contain a representative spectrum of constituents relevant to its biological activity (Supplementary).

The conditions of the extractions and the final concentrations of each product are shown in Table 1.

**Table 1:** Extraction conditions for each natural product and the final concentrations

Product	Extraction time	Solvent	Mass used for extraction (g)	Initial volume (ml)	Volume after filtration (ml)	Final volume (ml)	Concentration of the natural product (g/ml)
*Boswelia sacra (Burseraceae) (Frankincense)*	4 h	MeOH	5	50	37.0	12.5	0.40
*Calendula officinalis (Asteraceae) (Pot Marigold)*	1 h	80% EtOH	5	50	34.0	10.5	0.48
*Euphrasia officinalis (Orobanchaceae) (Eyebright)*	1d	70% MeOH	5	50	29.0	12	0.42
*Fagonia cretica (Zygophyllaceae) (Virgin’s mantle)*	2d	70%MeOH	5	50	34.0	22.5	0.22
*Hypericum perforatum (Hypericaceae)(St. John’s wort)*	5d	70% EtOH	5	50	31.5	9.5	0.53
*Matricaria chamomilla (Asteraceae)(Chamomile)*	5d	50% EtOH	5	50	29.5	9.5	0.53
*Moringa oleifera (Morigaceae) (Drumstick tree)*	1d	MeOH	5	50	38.0	5	1.00
*Morus alba (Moraceae) (White Mulberry)*	2d	95% EtOH	5	50	41.0	12	0.42
*Ocimum basilicum (Lamiaceae) (Basil)*	3d	70% EtOH	5	50	34.0	24	0.21
*Olea europaea (Oleaceae)(common Olive)*	4 h	80% EtOH	5	50	34.0	15	0.33
*Origanum vulgare (Lamiaceae)(Oregano)*	4w	40% EtOH	5	50	25.0	15	0.33
*Propolis (from bees)*	2d	70% EtOH	5	50	35.0	13	0.38
*Punica granatum (Lythraceae) (Pomegranate)*	2d	50% EtOH	5	50	38.0	20	0.25
*Rhodiola rosea (Crassulaceae) (Roseroot)*	4d	70% EtOH	5	50	33.5	9	0.56
*Ribes nigrum (Crossulariaceae) (Black currant)*	1d	50% EtOH	5	50	34.5	15.5	0.32
*Rosa canina (Rosaceae) (Rosehip)*	1 h	70% EtOH	5	50	37.5	9	0.56
*Rosmarinus officinalis (Lamiaceae)(Rosemary)*	1d	80% EtOH	5	50	31.0	20	0.25
*Salvia officinalis (Lamiaceae) (Sage)*	1d	70% MeOH	5	50	34.5	20	0.25
*Sideritis scardica (Lamiaceae)(Ironwort)*	1d	ΜeΟΗ	5	50	29.0	5	1.00
*Silybum marianum (Asteraceae) (Milk Thistle)*	4d	80% EtOH	5	50	37.0	8	0.63
*Thymus vulgaris (Lamiaceae) (Thyme)*	1d	70% EtOH	5	50	32.0	11	0.45
*Withania somnifera (Solanaceae) (Ashwagandha)*	1d	10% EtOH	5	50	36.0	9	0.56

All plant materials have been carefully stored and archived at the Laboratory of Pharmacology and Toxicology, Faculty of Veterinary Medicine, University of Thessaly, under appropriate labeling and documentation.

### Total phenolic content

The total phenolic content (TPC) of each sample was assayed via the Folin‒Ciocalteu (FC) method, as previously described [39]. Briefly, the plant extracts were mixed with FC reagent, and 509 mM Na_2_CO_3_ was added 2 min later. The samples were incubated for 2 h, after which their absorbance was determined at 765 nm via a UV‒VIS spectrophotometer (U-1900 Spectrophotometer, Hitachi, Ltd., Tokyo, Japan). The results are expressed in gallic acid equivalents. Each experiment was performed in triplicate, and the absorbance was corrected via blank solutions.

### Determination of antioxidant activity based on DPPH assay

DPPH^·^ scavenging was used as a part of the assessment of the antioxidant activity, as described in previous works [40]. Briefly, DPPH was dissolved in methanol, the samples were added at different concentrations, and the solutions were vigorously mixed. The final concentration of DPPH was 0.1 mM, and the reaction was carried out in the dark for 30 min. After the incubation period, the absorbance of each sample was determined at 517 nm. DPPH^·^ was used as a negative control, and methanol was used as a blank. Each experiment was conducted 3 times. The radical scavenging capacity (RSC) of the samples is expressed as the percentage of DPPH^·^ elimination, which is calculated via Eq. (1):

1 $$\text{\%RSC}=\frac{\text{Absorbance of control}-\text{Absorbance of sample }}{\text{Absorbance of control}}\text{x}100\%$$

### Estimation of antioxidant activity using the ABTS assay

The assay was performed as previously described [41]. A fresh aqueous solution of 7 mM ABTS was prepared, mixed with a solution of 2.45 mM potassium persulfate and stored for 16 h in the dark. The solution was then diluted with ethanol to achieve an absorbance value of 0.700 ± 0.05 at 734 nm. The plant extracts were added to the mixture, and the absorbance of the reaction solution was measured at 0 and 6 min. The calibration curve was generated with the results obtained using Trolox, and the results of the samples are expressed in Trolox equivalent antioxidant capacity (TEAC). Each sample was measured in triplicate, and the absorbance values were corrected using appropriate blank solutions.

### Cell culture and cell growth

K-562 cells were cultured under standard conditions as previously described [42]. The cells were grown in RPMI-1640 medium supplemented with 10% fetal bovine serum (FBS) and the antibiotics penicillin and streptomycin at 50 μg/ml and 100 μg/ml, respectively. The cultures were incubated at 37 °C under 5% CO_2_ in a humidified incubator. Cell growth was determined by counting the number of cells using a hemocytometer (Neubauer).

### Cytotoxicity assay

AlamarBlue (resazurin) is a commonly used indicator of cellular viability, based on the redox activity of metabolically active cells. The living cells reduce resazurin (blue, nonfluorescent) to resofurin (pink, fluorescent), allowing the evaluation of cytotoxicity caused by various treatments that cells undergo [43].

The cells were seeded in the wells of a 96-well plate. A total of 2.5 × 10^4^ cells per well were incubated with various concentrations of each extract. Following a 48 h incubation period, 20 μl of AlamarBlue was added, and the cells were then subjected to an additional 4 h incubation period. Fluorescence was measured via a fluorimeter (Cole-Parmer, United States) with excitation at 530 nm and emission at 590 nm. The percentage (%) of reduction of AlamarBlue was determined, and the *IC*_50_ of each extract was calculated. The assay was performed in triplicate.

### Bradford assay

The protein content of the cell extracts following incubation with or without the plant extracts for 48 h was determined via the Bradford assay [44]. Bradford reagent containing Coomassie blue G250 was used to interact with the proteins, and the absorbance was measured at 595 nm. The assay was performed as described in the manual of PanReac Applichem (Germany). To prepare the standard curve, bovine serum albumin was used. The cells were seeded in a 96-well plate, and the reagent was then added. The absorbance of each sample was measured using an infinite 200Pro plate reader (Tecan Trading AG, Switzerland). The assay was performed in triplicate.

### Caspase-3 activity assay

The caspase-3 activity assay was performed as indicated by the manufacturer’s recommendations (AnaSpec, Fremont, CA, USA). The cells were seeded in a 24-well plate at an initial density of 1 × 10^5^ cells/ml. The cells were incubated either with or without treatment with the respective plant extracts for 48 h and then collected and washed with PBS. The samples were subsequently lysed with RIPA lysis buffer and following centrifugation at 12,000 × g for 15 min, the supernatants were collected and mixed with reaction buffer (2% glycerol, 0.5 mM EDTA, 5 mM DTT, 100 mM HEPES, pH 7.5) and 100 μM apopain. The samples were incubated at 37 °C for 6 h, after which the absorbance was measured at 405 nm. The quantities utilized in this experiment corresponded to the concentration at which each of the extracts induced a 50% reduction in cell growth.

### RNA isolation

RNA was isolated according to the protocol developed by Zepeda and Verdonk (2022). In detail, cells were collected after a 24 h incubation period with each extract and lysed with cold lysis buffer containing 0.4 M ammonium thiocyanate, 0.8 M guanidine thiocyanate, 0.1 M sodium acetate (pH 5), 5% (v/v) glycerol, and 38% (v/v) phenol. Chloroform was added, and the supernatant was collected following centrifugation at 10,000 rpm for 5 min. Isopropanol was then added, and samples were incubated overnight at 4 °C. The RNA was collected, washed with 70% ethanol, dried and then resuspended in RNase-free water.

### Quantitative RT‒PCR

For reverse transcription PCR, a One-Step RT qPCR kit with SYBR Green (Bio-Rad Laboratories, USA) was used according to the instructions provided by the manufacturer. RT‒qPCR was carried out using a LightCycler 2.0 System (Roche Molecular Systems Inc., United States). Expression of *HO-1* (forward primer 5’-AAGTTCAAGCAGCTCTACCGCT-3’, reverse primer 5’-GGGCAGAATCTTGCACTTTGTTG-3’ [37]), *NQO1* (forward primer 5’-CGCAGACCTTGTGATATTCCAG-3’, reverse primer 5’-CGTTTCTTCCATCCTTCCAGG-3’ [47]), and *GCLC* (forward primer 5’- TGAGCATAGACACCATCATCAATG-3’, reverse primer 5’- TAGTTCTCCAGATGCTCTCTTCTT-3’ [37]) was measured. Gene expression was quantified in relation to the expression of the housekeeping gene *Actin Beta (ACTB)* [48] (forward primer 5’-AGAGCTACGAGCTGCCTGAC-3’, reverse primer 5’-AGCACTGTGTTGGCGTACAG-3’) [49]. The mean expression level ± standard deviation (SD) was calculated from 3 independent experiments. Eugenol was used as a positive control at 16.7 μΜ, as it has been shown to activate the Nrf2 pathway [50, 51].

### Statistical analysis

The results of experiments performed in triplicate are expressed as the means ± SDs. One-way ANOVA with post hoc analysis with Dunnett’s test was used to perform multiple comparisons (p < 0.05 was considered significant) after normality was assessed using the Shapiro‒Wilk test (a = 0.05). The statistical analysis was conducted using GraphPad Prism (version 8.0.1 for Windows, GraphPad Software, San Diego, California, USA).

## Results

### Total phenolic content and antioxidant activity

**Fig. 1:**
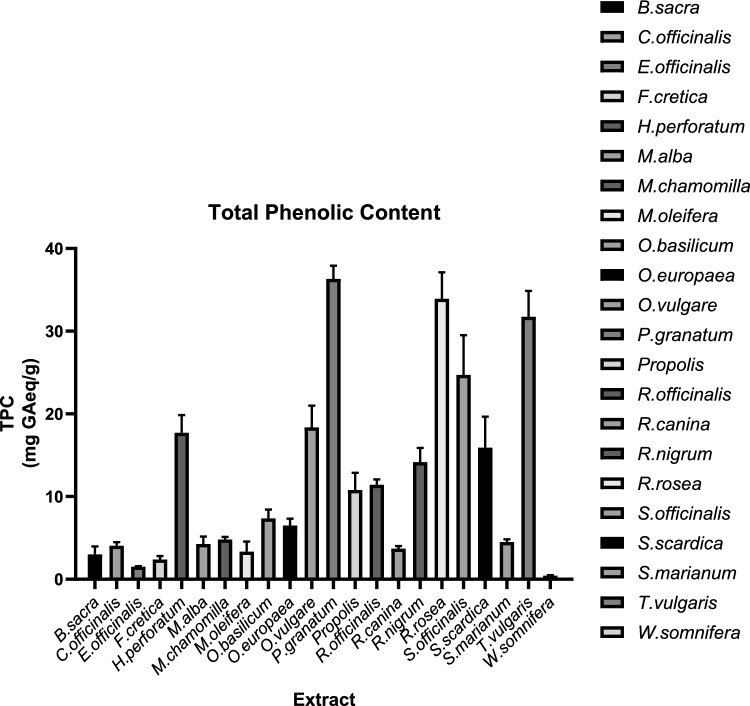
TPC of the plant extracts expressed in mg of gallic acid equivalents/g of plant used. The findings were derived from the utilization of various extracts at different concentrations and the expression of the results as gallic acid equivalents. The highest TPC was accredited to *P. granatum, R. rosea,* and *T. vulgaris*. The lowest TPC was exhibited by *W. somnifera*. The experiments were performed in triplicate, and the results are presented as the means ± SDs

The total phenolic content was determined using the modified Folin-Ciocalteu assay, which is compatible with 96-well plates and has a final volume of 200 μl. The results are expressed in mg/g gallic acid equivalent per gram of natural product. The results suggested that the total phenolic content (TPC) of the 22 natural extracts varied widely between 0.44 and 36.33 mg GAE/g of natural product. The ethanolic extracts from *Punica granatum, Rhodiola rosea*, and *Thymus vulgaris* showed the highest TPC values measured at 36.33 mg GAE/g, 33.91 mg GAE/g, and 31.74 mg GAE/g, respectively, with no statistically significant differences among them (p > 0.05). The extract of *Salvia officinalis* exhibited a TPC of 24.71 mg GAE/g, which was significantly different from that of the other extracts. Conversely, the extract from *Withania somnifera* had the lowest TPC, quantified at 0.44 mg GAE/g (Fig. 1, Table 2).

**Table 2:** TPC content and antioxidant activity of the extracts studied

Natural Product	TPC (mg GAE/g)	DPPH^·^ *IC*_*50*_ (mg/ml)	ABTS^·+^ *IC*_*50*_ (mg/ml)	ABTS_TEAC_ μmol Trolox eq/g
*Boswelia sacra (Frankincense)*	3.021 ± 0.942	ND	ND	ND
*Calendula officinalis* *(Pot Marigold)*	4.060 ± 0.420	0.820 ± 0.086	0.324 ± 0.017	29.387 ± 1.577
*Euphrasia officinalis (Eyebright)*	1.521 ± 0.072	4.276 ± 0.102	2.024 ± 0.026	4.700 ± 0.060
*Fagonia cretica (Virgin’s mantle)*	2.399 ± 0.404	1.920 ± 0.170	0.791 ± 0.009	12.033 ± 0.145
*Hypericum perforatum* *(St. John’s wort)*	17.730 ± 2.130	0.208 ± 0.016	0.097 ± 0.002	98.480 ± 1.992
*Matricaria chamomilla (Chamomile)*	4.810 ± 0.310	0.450 ± 0.013	0.216 ± 0.011	44.040 ± 2.223
*Moringa oleifera (Drumstick tree)*	3.35 ± 1.21	0.498 ± 0.03	0.233 ± 0.007	40.863 ± 1.313
*Morus alba (White Mulberry)*	4.252 ± 0.924	9.391 ± 0.486	0.3432 ± 0.05	2.773 ± 0.045
*Ocimum basilicum (Basil)*	7.368 ± 1.067	0.170 ± 0.007	0.117 ± 0.001	81.217 ± 0.774
*Olea europaea (common Olive)*	6.510 ± 0.820	0.587 ± 0.066	0.136 ± 0.009	70.247 ± 4.566
*Origanum vulgare (Oregano)*	18.370 ± 2.610	0.183 ± 0.005	0.057 ± 0.001	167.070 ± 3.736
*Propolis*	10.790 ± 2.10	0.187 ± 0.006	0.059 ± 0.003	161.703 ± 7.916
*Punica granatum (Pomegranate)*	36.328 ± 1.604	0.010 ± 0.001	0.004 ± 0.00003	2549.53 ± 25.32
*Rhodiola rosea (Roseroot)*	33.910 ± 3.220	0.141 ± 0.013	0.015 ± 0.001	646.673 ± 32.221
*Ribes nigrum (Black currant)*	14.170 ± 1.700	0.231 ± 0.005	0.046 ± 0.001	209.250 ± 6.857
*Rosa canina (Rosehip)*	3.690 ± 0.320	1.093 ± 0.082	0.305 ± 0.046	33.627 ± 2.686
*Rosmarinus officinalis (Rosemary)*	11.430 ± 0.650	0.189 ± 0.046	0.063 ± 0.003	151.183 ± 8.372
*Salvia officinalis (Sage)*	24.710 ± 4.800	0.177 ± 0.019	0.044 ± 0.003	44.700 ± 3.093
*Sideritis scardica (Ironwort)*	15.930 ± 3.720	0.495 ± 0.022	0.214 ± 0.015	92.133 ± 4.655
*Silybum marianum (Milk Thistle)*	1.630 ± 0.100	0.376 ± 0.057	0.103 ± 0.005	216.997 ± 14.557
*Thymus vulgaris (Thyme)*	31.740 ± 3.130	0.079 ± 0.006	0.045 ± 0.003	211.000 ± 12.324
*Withania somnifera (Ashwagandha)*	0.440 ± 0.070	7.547 ± 1.250	0.893 ± 0.012	10.657 ± 0.143

The results are expressed as the means ± SDs of 3 independent experiments. *B. sacra* did not exhibit any potent scavenging activity for the free radicals DPPH and ABTS. Thus, its *IC*_50_ is nondetermined (ND)

To assess the antioxidant activities of the examined extracts, DPPH^•^ and ABTS^•+^ assays were performed.

**Fig. 2:**
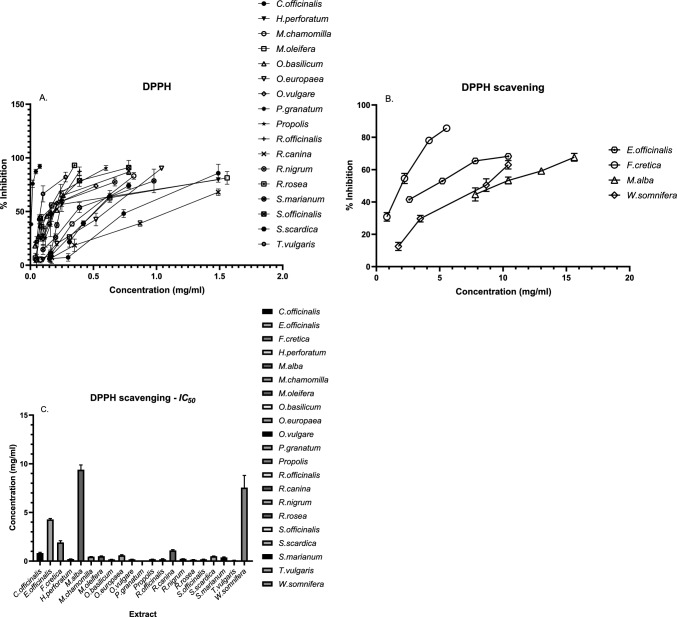
**A, B** Effects of various concentrations of each sample on the inhibition of DPPH^•^ free radicals. The impact of *B. sacra* extract could not be determined. **C** The *IC*_50_ of each sample is presented here. Three independent experiments were performed, and the results are expressed as the means ± SDs

The ability of the plant extracts to scavenge DPPH radicals, as indicated by their *IC*_50_ (mg/ml) values, showed considerable variation. *Punica granatum* demonstrated the most potent activity, with an *IC*_50_ of 0.01 mg/ml, reflecting strong antioxidant potential. In contrast, *Morus alba* and *Withania somnifera* had relatively high *IC*_50_ values of 9.391 mg/ml and 7.547 mg/ml, respectively, suggesting limited DPPH^•^ radical scavenging. The DPPH^•^ scavenging capacity of the extract obtained from *Boswellia sacra* could not be determined (Fig. 2).

**Fig. 3:**
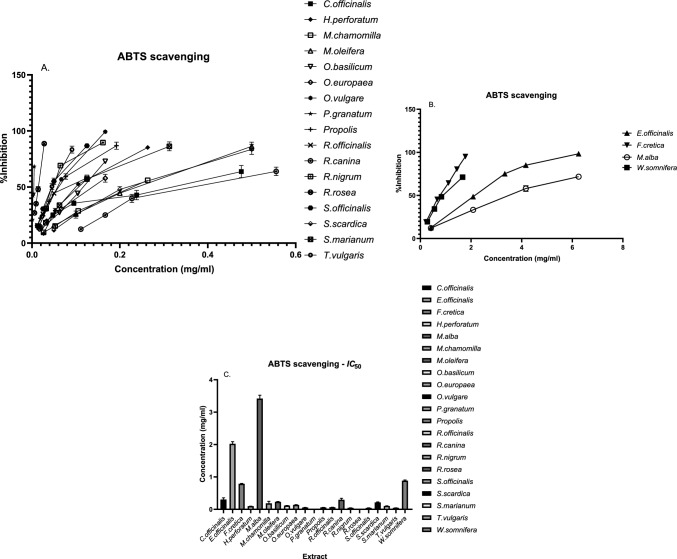
**A, B** The impact of each sample on ABTS^·+^ is demonstrated in relation to its concentration. The effect of *B. sacra* on the ABTS radical could not be determined. **C** The concentration of each extract that resulted in 50% inhibition of ABTS^·+^ expressed in mg/ml. The results are expressed as the means ± SDs from three independent experiments

The antioxidant capacity of the samples was also assessed using the ABTS^•+^ radical scavenging method. To determine the half-maximal inhibitory concentration (*IC*_50_), various concentrations of each sample were tested. The antioxidant potential was quantified in terms of Trolox equivalent antioxidant capacity (expressed as μmol Trolox/g of product and as mg/ml). The *P. granatum* extract presented the highest ABTS^•+^ scavenging activity, reflected by the lowest *IC*_50_ value (0.004 mg/ml, 2549.53 μmol Trolox eq/g), followed by *R. rosea,* with values of 0.015 mg/ml and 646.67 μmol Trolox eq/g, respectively. In contrast, *Euphrasia officinalis* displayed the least efficient ABTS^•+^ scavenging capability, with values of 2.020 mg/ml and 4.70 μmol Trolox eq/g. Additionally, the ABTS^•+^ scavenging activity of the *Boswellia sacra* extract could not be determined; thus, it was labeled nondetermined (ND). The results are illustrated in Fig. 3.

### Alamar blue cytotoxicity assay

**Fig. 4:**
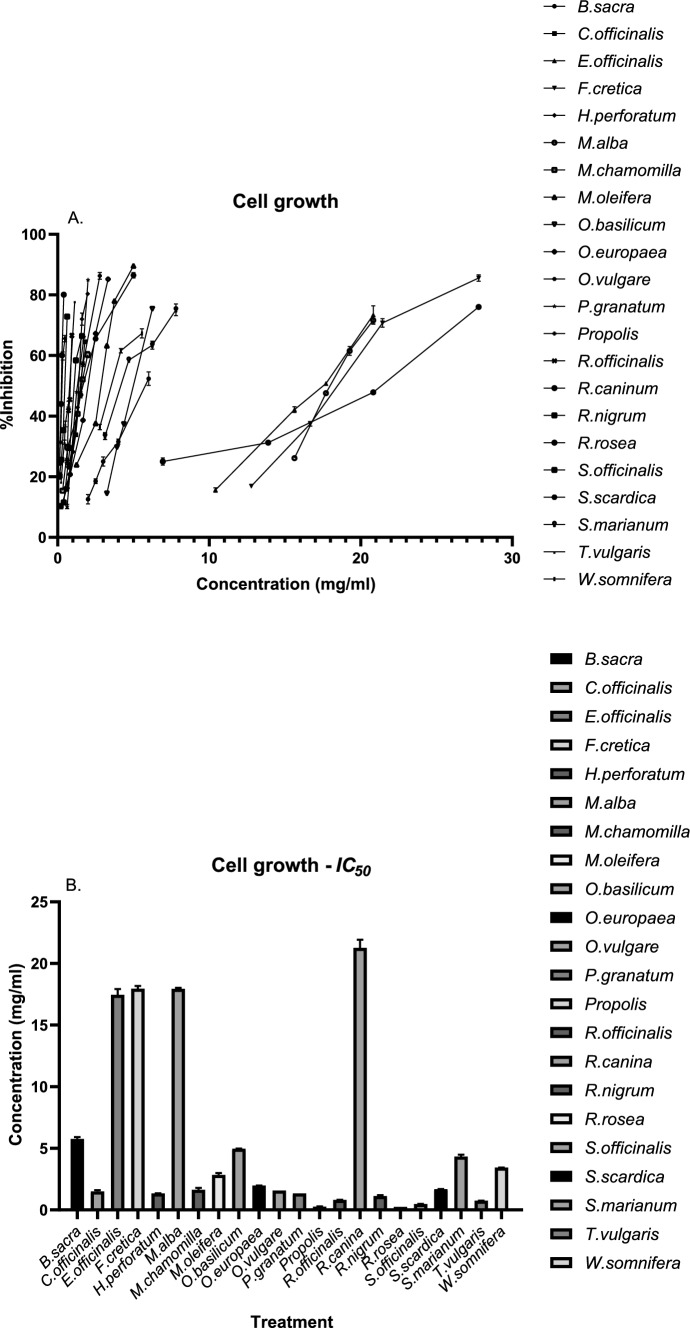
**A** Inhibition of K-562 cell growth after treatment with various plant extracts. **B** *IC*_50_ values of various plant extracts in K-562 cells, indicating the concentration at which cell viability was reduced by 50%. The values are presented in mg/ml. The experiments were performed in triplicate, and the results are shown as the means ± SDs

The cells were seeded in 96-well plates at an initial density of 2.5 × 10^4^ cells/well. The samples were incubated with the plant extracts for 48 h before the cytotoxicity of each sample was determined. Different concentrations of each extract were applied due to their varying levels of cytotoxicity. The impact of each sample on cell viability was determined through the fluorescence emitted at 590 nm after excitation at 530 nm using the resazurin assay. Living cells reduce resazurin, and a fluorescent product is formed [43]. Cell viability was affected in a concentration-dependent manner, and the *IC*_*50*_ of each sample was determined and expressed in mg/ml. The greatest cytotoxic effect was observed in *Rhodiola rosea,* which exhibited the lowest *IC*_50_ value (0.25 mg/ml), followed by Propolis (0.28 mg/ml) and *Salvia officinalis* (0.48 mg/ml). The lowest cytotoxicity was observed with the extracts of *Rosa canina* (21.27 mg/ml), *Fagonia cretica* (17.95 mg/ml)*, Morus alba* (17.93 mg/ml) and *Euphrasia officinalis* (17.45 mg/ml). The *IC*_*50*_ values for each natural product along with their effects on caspase-3 activity (see below) are presented in Fig. 4.

### Caspase-3 activity assay

**Fig. 5:**
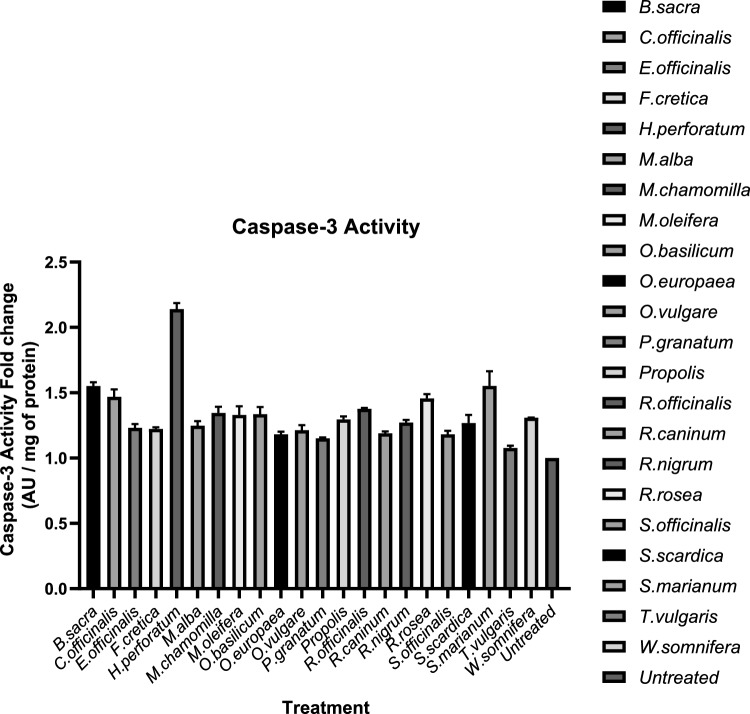
Fold change in caspase-3 activity between treated and untreated cells. Caspase-3 activity is expressed as absorbance units (AUs) per μg of protein. All the treatments except the *T. vulgaris* extract resulted in statistically significant differences compared with the untreated cells. The results are representative of three experiments, and the values are reported as the means ± SDs

Each extract was tested at a concentration corresponding to its *IC*_50_ value, in triplicate. Caspase-3 activity is expressed as the fold change between treated and untreated cells. K-562 cells treated with *Hypericum perforatum* presented a marked increase in caspase-3 activity (2.14-fold) which was statistically significant compared with that of the other treatments and that of the untreated cells. In contrast, caspase-3 activity in *Thymus vulgaris*-treated cells did not differ significantly from that in untreated cells (Fig. 5). Among the plant derivatives that were also analyzed for their effect on the expression of Nrf2 target genes, *Salvia officinalis* induced a 1.18-fold increase in caspase-3 activity, *Euphrasia officinalis* resulted in a 1.23-fold increase, whereas *Withania somnifera, Moringa oleifera, Rosmarinus officinalis* and *Rhodiola rosea* caused 1.31-, 1.33-, 1.38- and 1.46-fold increases, respectively.

### Plant extracts affect Nrf-2-responsive genes

**Fig. 6:**
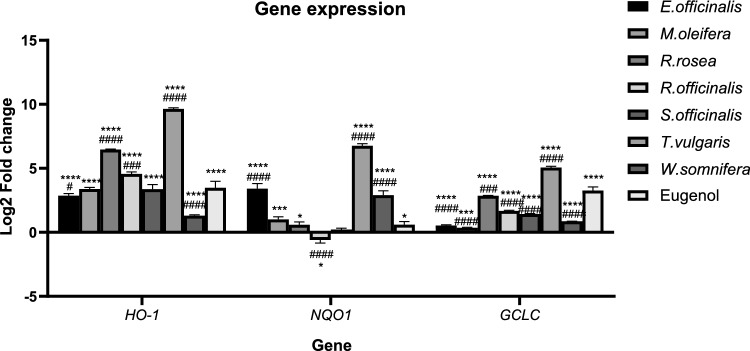
Logarithmic fold changes in *HO-1, NQO1* and *GCLC* expression after each treatment. The gene expression of untreated cells was 0 Log_2_FC. Eugenol (16.7 μM) was used as a positive control for Nrf2 target gene expression. Three independent experiments were performed, and the results are presented as the means ± SDs. ^#^p < 0.05, ^##^p < 0.01, ^###^p < 0.001, ^####^p < 0.0001 vs eugenol-treated cells (for the corresponding gene). *p < 0.05, **p < 0.01, ***p < 0.001, ****p < 0.0001 vs untreated cells (for each corresponding gene). The Log2FC of untreated cells was equal to zero (0)

The quantification of the expression levels of three genes regulated by Nrf2 was performed for each treatment following a 24 h incubation of the cells with the plant extracts. The housekeeping gene *ACTB* was used as a reference for normalization of Nrf2 target gene expression. The relative change in RNA expression compared with that in the control group was expressed as the log2-fold change (log2FC). The expression of *HO-1* increased after every treatment. Compared with that of the untreated cells, the most significant Log2FC of *HO-1* gene expression was caused by *Thymus vulgaris* (9.65), followed by *Rhodiola rosea* (6.47), *Rosmarinus officinalis* (4.59), Eugenol (3.49), *Salvia officinalis* (3.37), *Moringa oleifera* (3.38), *Euphrasia officinalis* (2.86), and *Withania somnifera* (1.30). *HO-1* expression in cells treated with *Moringa oleifera* or *Salvia officinalis* did not significantly differ from *HO-1* expression in cells treated with eugenol, whereas the other treatments induced significantly different expression levels. *NQO1* expression increased (log2FC > 0) following treatment with *Thymus vulgaris* (6.75), *Euphrasia officinalis* (3.41), *Withania somnifera* (2.9), *Moringa oleifera* (1.02), Eugenol (0.6), *Rhodiola rosea* (0.59) or *Salvia officinalis* (0.22). In contrast, the downregulation of *NQO1* (log2FC < 0) was observed when the cells were treated with *Rosmarinus officinalis* (− 0.6). *NQO1* expression in eugenol-treated cells was similar to that in cells treated with *Moringa oleifera, Rhodiola rosea* or *Salvia officinalis*. The other treatments significantly altered *NQO1* expression compared with the expression observed in cells treated with eugenol. Similarly, the expression of *GCLC* was upregulated by all the treatments, with the highest expression found in the *Thymus vulgaris-*treated cells (5.06), followed by the cells treated with eugenol (3.27), *Rhodiola rosea* (2.85), *Rosmarinus officinalis* (1.67), *Salvia officinalis* (1.43), *Withania somnifera* (0.86), *Euphrasia officinalis* (0.54), and *Moringa oleifera* (0.35). Statistically significant differences in the expression levels of *GCLC* were found between the cells treated with eugenol and the cells treated with natural products. The expression of Nrf2 target genes was affected in most instances, although this was not the case for treatment with *Salvia officinalis*, where *NQO1* expression was not significantly different from the *NQO1* expression that was observed in untreated cells (Fig. 6, Table 4).

## Discussion

Several plant extracts have been assessed for their ability to scavenge free radicals and, subsequently, for their effects on the death and apoptosis of K-562 cells. There is a strong correlation between the total phenolic content of plant extracts and their antioxidant activity [52]. While most of the extracts had distinctive TPC and antioxidant activities, the extract from *Punica granatum* peel exhibited the highest TPC, along with the strongest DPPH^·^ and ABTS^·+^ radical neutralizing capacities. The extract of *R. rosea* displayed potent radical neutralizing abilities in both the DPPH^·^ and ABTS^·+^ assays. Notably, *Rhodiola imbricata* has been shown to decrease the growth of K-562 cells, and salidroside (a chemical constituent of *R. rosea*) has been associated with the inhibition of caspase-3 [53]. In our study, both *P. granatum* and *R. rosea* increased caspase-3 activity and had cytotoxic effects on K-562 cells, consistent with the behavior observed for most of the natural products tested.

Caspase-3 is recognized as an apoptosis mediator, and although certain caspases regulate alternative cell death pathways, such as pyroptosis, ferroptosis operates independently of caspase activity [54]. Caspase-3 is a pivotal component in the orchestration of the apoptosis pathway [55]. Most of the natural products tested significantly increased caspase-3 activity in K-562 cells. Among them, *Hypericum perforatum* extract induced the greatest increase compared with the control (Figs. 4, 5, Table 3). In contrast, treating cells with thyme extract did not significantly alter caspase-3 activity, despite exhibiting cytotoxicity levels comparable to other extracts. The highest cytotoxic potential was observed with *R. rosea* and propolis extracts, while treatment with *Salvia officinalis, T. vulgaris* or *Rosmarinus officinalis* also resulted in strong cytotoxic activity, which is in agreement with previous studies [56, 57]. Notably, although the extract of *T. vulgaris* did not significantly alter the caspase-3 activity of K-562 cells, it produced one of the lowest *IC*_50_ values observed, highlighting its efficacy against K-562 cells. *Moringa oleifera*'s methanolic extract has displayed cytotoxic effects on prostate cancer cells (PC-3), but when it was administered to K-562 cells, it inhibited their proliferation without inducing apoptosis [58, 59]. *Withania somnifera* is known to contain withanolide D, a compound that is cytotoxic and known to inhibit the proliferation of K-562 cells [60].

**Table 3:** Inhibition of cell growth when treated with each natural product in relation to the control group, provided as the IC_50_ of the natural products after 48 h of incubation

Natural product	*IC*_*50*_ (mg/ml)	Caspase-3 activity increase ratio to untreated cells
*Boswelia sacra (Frankincense)*	5.76 ± 0.15	1.55 ± 0.03
*Calendula officinalis (Pot Marigold)*	1.51 ± 0.10	1.47 ± 0.06
*Euphrasia officinalis (Eyebright)*	17.45 ± 0.48	1.23 ± 0.03
*Fagonia cretica (Virgin’s mantle)*	17.95 ± 0.22	1.22 ± 0.01
*Hypericum perforatum (St. John’s wort)*	1.34 ± 0.02	2.14 ± 0.05
*Matricaria chamomilla (Chamomile)*	1.63 ± 0.15	1.35 ± 0.05
*Moringa oleifera (Drumstick tree)*	2.84 ± 0.16	1.33 ± 0.07
*Morus alba (White Mulberry)*	17.93 ± 0.08	1.25 ± 0.03
*Ocimum basilicum (Basil)*	4.96 ± 0.01	1.34 ± 0.06
*Olea europaea (common Olive)*	1.98 ± 0.01	1.18 ± 0.02
*Origanum vulgare (Oregano)*	1.57 ± 0.01	1.21 ± 0.04
*Propolis*	0.28 ± 0.01	1.38 ± 0.08
*Punica granatum (Pomegranate)*	1.34 ± 0.01	1.15 ± 0.07
*Rhodiola rosea (Roseroot)*	0.25 ± 0.01	1.45 ± 0.03
*Ribes nigrum (Black currant)*	1.13 ± 0.06	1.27 ± 0.02
*Rosa canina (Rosehip)*	21.27 ± 0.65	1.19 ± 0.01
*Rosmarinus officinalis (Rosemary)*	0.82 ± 0.01	1.38 ± 0.01
*Salvia officinalis (Sage)*	0.48 ± 0.01	1.18 ± 0.03
*Sideritis scardica (Ironwort)*	1.69 ± 0.01	1.26 ± 0.06
*Silybum marianum (Milk Thistle)*	4.33 ± 0.15	1.55 ± 0.11
*Thymus vulgaris (Thyme)*	0.75 ± 0.01	1.08 ± 0.02
*Withania somnifera (Ashwagandha)*	3.43 ± 0.01	1.31 ± 0.01

The experiments were performed three times, and the results are expressed as the means ± SDs

We selected plant extracts with different potencies to evaluate their effects on Nrf2 target genes. In *S. officinalis*-treated cells, *HO-1* and *GCLC* but not *NQO1* were upregulated. In cells treated with *R. officinalis, HO-1* and *GCLC* were upregulated, whereas *NQO1* was downregulated. However, in the cells treated with *M. oleifera, W. somnifera, E. officinalis, R. rosea* or *T. vulgaris,* all three genes were upregulated. The application of rosemary extract resulted in increased caspase-3 activity and increased *GCLC* and *HO-1 expression*, while it concurrently suppressed *NQO1* expression. This observation contradicts earlier findings showing that *R. officinalis* extracts upregulate *NQO1* expression [61]. *M. oleifera* has been previously reported to increase the expression of the Nrf2 target genes *HO-1* and *NQO1* [62]. *W. somnifera* is recognized as a potent inducer of the Nrf2 pathway and has been shown to specifically augment the expression of *HO-1* [63]. *Salvia officinalis* contains bioactive compounds that are competent in activating specific Nrf2 target genes, notably *NQO1* and *GCLC* [64]. Various phytochemicals (such as sulforaphane and curcumin) act as Michael acceptors to the highly reactive cysteines of Keap1, thus inhibiting its activity and activating the Nrf2 transcription factor [65]. The highest mRNA levels of the 3 genes were detected in cells treated with *T. vulgaris*. Thyme has previously been studied for its protective effects on skin following UVB exposure, during which activation of the Nrf2 pathway was observed [66]. Moreover, *R. rosea* has been shown to activate the Nrf2 signaling cascade, leading to the upregulation of *NQO1, HO-1*, and *GCLC*. One of the main compounds in *Rhodiola*, salidroside, has been scientifically demonstrated to be a key player in inducing the Nrf2 signaling pathway [67]. The complex modulation of these molecular pathways by various plant extracts provides a compelling narrative for potential therapeutic interventions (Table 4, Fig. 6).

**Table 4:** Expression of Nrf2 target genes (*HO-1, NQO1, and GCLC*) after a 24 h incubation period of K-562 cells; the expression of the natural products is expressed as the log2-fold change

	*HO-1* (Log_2_ Fold Change)	*NQO1* (Log_2_ Fold Change)	*GCLC* (Log_2_ Fold Change)
*Euphrasia officinalis*	2.86 ± 0.16	3.41 ± 0.4	0.54 ± 0.05
*Moringa oleifera*	3.38 ± 0.12	1.02 ± 0.19	0.35 ± 0.05
*Rhodiola rosea*	6.47 ± 0.04	0.59 ± 0.23	2.85 ± 0.01
*Rosmarinus officinalis*	4.59 ± 0.06	− 0.6 ± 0.24	1.67 ± 0.06
*Salvia officinalis*	3.37 ± 0.36	0.22 ± 0.10	1.43 ± 0.04
*Thymus vulgaris*	9.65 ± 0.09	6.75 ± 0.17	5.06 ± 0.09
*Withania somnifera*	1.30 ± 0.07	2.90 ± 0.34	0.86 ± 0.02
Eugenol	3.49 ± 0.50	0.60 ± 0.24	3.27 ± 0.28

Eugenol (16.70 μΜ) was used as a positive control

*Euphrasia officinalis* has been shown to have antioxidant and anti-inflammatory effects on human corneal cells and dermal fibroblasts [68, 69]. Eyebright affects the NF-κΒ signaling pathway, resulting in the suppression of NF-κΒ and therefore its anti-inflammatory effects [68]. *E. officinalis* has also been shown to upregulate Nrf2 target genes (Table 4, Fig. 6). In fact, Nrf2 exerts complementary to its antioxidant effects, direct anti-inflammatory actions through its binding to the promoters of key inflammatory genes, promoting their transcriptional suppression [70]. When extracted with heptane, *Euphrasia officinalis* has demonstrated anti-proliferative and cytotoxic effects on human corneal cells. In contrast, when extracted with ethanol or ethyl acetate, this anti-proliferative activity was not detected [69]. In our work, *E. officinalis* extracted with 70% aqueous methanol was associated with a decrease in the viability of K-562 cells and a corresponding increase in caspase-3 activity.

Although Nrf2 is often associated with chemoresistance in established cancer cell lines, its activation can also serve as a cellular defense mechanism against oxidative stress and DNA damage, thereby contributing to cancer prevention. In this study, Nrf2 activation was evaluated not as a therapeutic target in K-562 cells, but rather as a functional readout to assess the chemopreventive potential of the plant extracts. The simultaneous induction of apoptosis observed in our study suggests that certain natural products may exhibit a dual role and the involvement of parallel pathways.

A limitation of this study is the absence of chromatographic or spectral characterization of the plant extracts. Although the plants investigated are commonly used in ethnomedicine and have been chemically characterized in prior studies, it is acknowledged that extraction conditions, origin, and plant part may result in distinct phytochemical profiles. Therefore, the lack of direct chemical analysis in the present study restricts the attribution of the observed bioactivity to specific constituents.

Although chemical characterization of the extracts was not performed in this study, the correlation observed between the total phenolic content (TPC) and antioxidant or cytotoxic activity suggests that phenolic compounds may contribute substantially to the bioactivity of certain plant extracts. It has been reported that phenolic constituents, such as flavonoids, phenolic acids and tannins, are capable of exerting strong antioxidant effects and modulating apoptosis in cancer cells, including leukemic cells [71, 72]. For instance punicalagin from *Punica granatum* has been associated with caspase activation and ROS-mediated apoptosis in leukemia models [73]. Similarly, salidroside, found in *R. rosea*, has been reported to modulate caspase-3 activity and inhibit proliferation in K-562 cells [74]. However, the involvement of other phytochemicals, such as alkaloids, terpenoids or iridoids, cannot be excluded. For example, terpinolene, a monoterpene with strong antioxidant and pro-apoptotic properties, and withanolides, the steroidal lactones found in *Withania somnifera* known for their cytotoxicity and Nrf2-inducing potential, may also contribute to the observed bioactivities [56, 60, 75]. Therefore, we acknowledge that further studies are necessary to confirm which compounds are responsible for the observed biological effects.

This study revealed that each plant extract exerted a suppressive effect on cellular proliferation, albeit potentially through various mechanistic pathways. Plant extracts that have a low *IC*_50_ value in cellular growth assays are promising sources for the extraction of bioactive compounds. These compounds might subsequently serve as precursors or building blocks for the development of novel anticancer therapeutic agents [76].

## Conclusion

This study provides a comprehensive evaluation of 22 natural product extracts in terms of their antioxidant activity, cytotoxic effects, and capacity to modulate Nrf2-regulated gene expression in human K-562 leukemia cells. Several extracts exhibited strong radical scavenging activity and induced apoptosis via caspase-3 activation, reflecting their pro-oxidant and cytotoxic properties in this cancer model. Our findings suggest that plant extracts exhibit growth-inhibitory properties via different mechanisms. Notably, a subset of extracts, including *T. vulgaris, R. rosea* and *E. officinalis*, significantly upregulated Nrf2 target genes, such as *HO-1, NQO1* and *GCLC*, indicating that their biological activity is at least partly mediated through activation of the Nrf2 signaling pathway.

These findings underscore the therapeutic potential of selected natural products and their compounds as redox modulators in the treatment of malignancies. Furthermore, the different responses elicited by the extracts highlight the complexity of phytochemical bioactivity and the necessity for mechanistic evaluations beyond standard antioxidant assays. Future research is needed to isolate and characterize the active phytochemicals responsible for these effects and to evaluate their efficacy in models of leukemia and oxidative stress-related diseases. In particular, the dual antioxidant and anti-inflammatory profile of *Euphrasia officinalis* merits additional investigation as a Nrf2 modulator.

## Supplementary Information

Below is the link to the electronic supplementary material.

Supplementary file1 (DOCX 43 KB)

## Data Availability

No datasets were generated or analyzed during the current study.

## Abbreviations

ABTS: 2,2’-Azino-bis(3-ethylbenzothiazoline-6-sulfonic acid)
CML: Chronic myeloid leukemia
DMSO: Dimethyl sulfoxide
DPPH: 2,2-Diphenyl-1-picrylhydrazyl
DTT: Dithiothreitol
EDTA: Ethylenediaminetetraacetic acid
EtOH: Ethanol
FBS: Fetal bovine serum
FC: Folin-Ciocalteu
GAE: Gallic acid equivalents
GCLC: Glutamate-cysteine ligase catalytic subunit
HO-1: Heme oxygenase 1
Keap1: Kelch-like ECH-associated protein 1
MeOH: Methanol
NP: Natural product
NQO1: NAD(P)H quinone dehydrogenase 1
Nrf2: Nuclear factor erythroid 2-related factor 2
PBS: Phosphate-buffered saline
RSC: Radical scavenging capacity
RT‒qPCR: Quantitative reverse transcription polymerase chain reaction
TEAC: Trolox equivalent antioxidant capacity
TPC: Total phenolic content
