# Supplementary material for: Natural Nrf2 activators modulate antioxidant gene expression and apoptosis in leukemic K-562 cells
Source: Med Oncol. 2025 Jul 31;42(9):396. doi: 10.1007/s12032-025-02946-4 (PMC12313825; doi:10.1007/s12032-025-02946-4)
Supplement: Supplementary file 1 — Supplementary file1 (DOCX 43 KB) [file 12032_2025_2946_MOESM1_ESM.docx]

**Supplementary material: Justification of Extraction Solvents Used for Each Plant Material**

The extraction protocols employed in this study were selected based on literature, aiming to maximize the recovery of bioactive compounds using appropriate hydroethanolic or methanolic mixtures. Below is a summary of the selected solvent systems and supporting references for each plant extract:

**10% Ethanol**

- *Withania somnifera*: Extracted using 10% aqueous ethanol at room temperature, which has been previously reported to effectively yield withanolides and other bioactive constituents [1].

**40% Ethanol**

- *Origanum vulgare*: Successfully extracted using 40% ethanol, enabling recovery of phenolic compounds and supporting the appropriateness of this solvent system [2].

**50% Ethanol**

- *Matricaria chamomilla*: A 50% hydroethanolic solution has been previously used to extract its bioactive constituents effectively [3].
- *Punica granatum*: Extracted with 50% ethanol, which is effective for recovering phenolic and antioxidant compounds [4].
- *Ribes nigrum*: Extraction with 50% ethanol has been optimized in previous studies for obtaining phenolic-rich extracts with antioxidant and cytotoxic properties [5].

**70% Ethanol**

- *Hypericum perforatum*: Extracted using 70% ethanol, which is widely cited as effective for recovering hypericin, hyperforin, chlorogenic acids, and flavonoids such as quercetin and rutin [6].
- *Ocimum basilicum*: Extracted with 70% ethanol to enhance the yield of diverse phytochemicals [7].
- *Propolis*: Extracted using 70% ethanol, a well-established method for obtaining flavonoids and phenolic acids [8].
- *Rhodiola rosea*: Hydroethanolic solutions at 70% are commonly used to solubilize phenolic compounds, flavonoids, and salidroside [9].
- *Rosa canina*: 70% ethanol has been shown to recover both hydrophilic and lipophilic phytochemicals [10].
- *Thymus vulgaris*: 70% ethanol is reported to efficiently extract phenolic compounds with biological activity [11].

**80% Ethanol**

- *Calendula officinalis*: 80% ethanol has demonstrated high efficiency in extracting phenolic and flavonoid compounds [12]*.*
- *Olea europaea* (olive leaf): 80% ethanol was chosen based on studies evaluating its efficiency in extracting oleuropein [13].
- *Rosmarinus officinalis*: 80% ethanol has been used to isolate a wide range of bioactive compounds [14].
- *Silybum marianum*: Commonly extracted using 80% ethanol to yield flavonolignans and phenolics with antioxidant activity and high bioactive content [15,16].

**95% Ethanol**

- *Morus alba*: Extracted with 95% ethanol, which effectively isolates polyphenolic fractions and antioxidant compounds [17].

**70% Methanol**

- *Fagonia cretica*: 70% methanol extracted a broad spectrum of phytochemical classes including flavonoids, terpenoids, coumarins, and steroids [18].
- *Salvia officinalis*: 70% methanol effectively extracts polyphenols, flavonoids, and phenolic acids [19].

**100% Methanol**

- *Boswellia sacra*: Methanol is used to extract boswellic acids and other active compounds with antioxidant and cytotoxic properties [20,21].
- *Euphrasia officinalis*: 100% methanol extracts high levels of several key bioactive compounds—including caffeic and ferulic acid derivatives, while 100% water extracts contained additional constituents not present in the methanolic fraction. A 70% methanol compromise was used in our study [22].
- *Moringa oleifera*: Methanolic extraction yields flavonoids, alkaloids, and phenolics and exhibits antimicrobial and antioxidant activities [23].
- *Sideritis scardica*: Methanol has been shown to extract a rich profile of phenolics, with strong antioxidant potential [24].

**Extraction Time Notes**

Extraction times were tailored to each plant based on matrix composition. Soft tissues (e.g., leaves and flowers) were extracted over shorter durations, while tougher or larger powder particle sizes required extended maceration. This strategy aligns with established findings that particle size and extraction time significantly influence the yield [25].

Supplementary material references

1. Dhanani T, Shah S, Gajbhiye NA, Kumar S. Effect of extraction methods on yield, phytochemical constituents and antioxidant activity of Withania somnifera. Arab J Chem [Internet]. 2017;10:S1193–9. Available from: http://dx.doi.org/10.1016/j.arabjc.2013.02.015

2. Hambardzumyan S, Sahakyan N, Petrosyan M, Nasim MJ, Jacob C, Trchounian A. Origanum vulgare L. extract-mediated synthesis of silver nanoparticles, their characterization and antibacterial activities. AMB Express [Internet]. 2020;10. Available from: https://doi.org/10.1186/s13568-020-01100-9

3. M. Moricz A, Szarka S, G. Ott P, B. Hethelyi E, Szoke E, Tyihak E. Separation and Identification of Antibacterial Chamomile Components Using OPLC, Bioautography and GC-MS. Med Chem (Los Angeles). 2012;8:85–94.

4. Pagliarulo C, De Vito V, Picariello G, Colicchio R, Pastore G, Salvatore P, et al. Inhibitory effect of pomegranate (Punica granatum L.) polyphenol extracts on the bacterial growth and survival of clinical isolates of pathogenic Staphylococcus aureus and Escherichia coli. Food Chem [Internet]. 2016;190:824–31. Available from: http://dx.doi.org/10.1016/j.foodchem.2015.06.028

5. Jia N, Xiong YL, Kong B, Liu Q, Xia X. Radical scavenging activity of black currant (Ribes nigrum L.) extract and its inhibitory effect on gastric cancer cell proliferation via induction of apoptosis. J Funct Foods [Internet]. 2012;4:382–90. Available from: http://dx.doi.org/10.1016/j.jff.2012.01.009

6. Ion V, Ielciu I, Cârje AG, Muntean DL, Crişan G, Păltinean R. Hypericum spp.—An Overview of the Extraction Methods and Analysis of Compounds. Separations. 2022;9:1–21.

7. Nadeem HR, Akhtar S, Sestili P, Ismail T, Neugart S, Qamar M, et al. Toxicity, Antioxidant Activity, and Phytochemicals of Basil (Ocimum basilicum L.) Leaves Cultivated in Southern Punjab, Pakistan. Foods. 2022;11:1–13.

8. Liaudanskas M, Kubilienė L, Žvikas V, Trumbeckaitė S. Comparison of Ethanolic and Aqueous-Polyethylenglycolic Propolis Extracts: Chemical Composition and Antioxidant Properties. Evidence-based Complement Altern Med. 2021;2021.

9. Sęczyk Ł, Sugier D, Dervişoğlu G, Özdemir FA, Kołodziej B. Phytochemical profile, in vitro bioaccessibility, and anticancer potential of golden root (Rhodiola rosea L.) extracts. Food Chem. 2023;404.

10. Fetni S, Bertella N, Ouahab A, Martinez Zapater JM, De Pascual-Teresa Fernandez S. Composition and biological activity of the Algerian plant Rosa canina L. by HPLC-UV-MS. Arab J Chem [Internet]. 2020;13:1105–19. Available from: https://doi.org/10.1016/j.arabjc.2017.09.013

11. El-Newary SA, Shaffie NM, Omer EA. The protection of Thymus vulgaris leaves alcoholic extract against hepatotoxicity of alcohol in rats. Asian Pac J Trop Med. 2017;10:361–71.

12. Messina CM, Troia A, Arena R, Manuguerra S, Ioannou T, Curcuraci E, et al. Species-specific antioxidant power and bioactive properties of the extracts obtained from wild mediterranean Calendula Spp. (Asteraceae). Appl Sci. 2019;9:1–13.

13. Yateem H, Afaneh I, Al-Rimawi F. Optimum Conditions for Oleuropein Extraction from Olive Leaves. Int J Appl Sci Technol. 2014;4:153–7.

14. Abo El-Maati MF, Gedamy GM, Hefnawy HT, Awad AE, EL-Maghraby LMM. Chemical Characterization of Rosmarinus officinalis L.Hydrodistillation of By-Products, Evaluating Their Antioxidant, and Anticancer Activities. Egypt J Chem. 2025;68:369–79.

15. Kalinowska M, Płońska A, Trusiak M, Gołębiewska E, Gorlewska-Pietluszenko A. Comparing the extraction methods, chemical composition, phenolic contents and antioxidant activity of edible oils from Cannabis sativa and Silybum marianu seeds. Sci Rep [Internet]. 2022;12:1–16. Available from: https://doi.org/10.1038/s41598-022-25030-7

16. Chikhoune A, Ghazi A, Adjadj F. Bioactive Potential of Milk Thistle (Sylibum marianum) Seeds and Applicability of Its Edible Oil in Food Processing. 2024;2.

17. Dugo P, Donato P, Cacciola F, Germanò MP, Rapisarda A, Mondello L. Characterization of the polyphenolic fraction of Morus alba leaves extracts by HPLC coupled to a hybrid IT-TOF MS system. J Sep Sci. 2009;32:3627–34.

18. Tunio QUN, Rafiq M, Tunio AA, Qureshi AS, Charan TR, Bhutto MA, et al. Determination of Phytochemicals, Antimicrobial, Antioxidant and Allelopathic Effects of Fagonia cretica L., collected from Jamshoro, Pakistan. Yuz Yil Univ J Agric Sci. 2022;32:785–94.

19. Hrebień-Filisińska AM, Tokarczyk G. The Use of Ultrasound-Assisted Maceration for the Extraction of Carnosic Acid and Carnosol from Sage (Salvia officinalis L.) Directly into Fish Oil. Molecules. 2023;28.

20. Alipanah H, Zareian P. Anti-cancer properties of the methanol extract of boswellia serrata gum resin: Cell proliferation arrest and inhibition of angiogenesis and metastasis in BALB/c mice breast cancer model. Physiol Pharmacol. 2018;22:183–94.

21. Alharbi SA, Asad M, Abdelsalam KEA, Ibrahim MA, Chandy S. Beneficial Effect of Methanolic Extract of Frankincense (Boswellia Sacra) on Testis Mediated through Suppression of Oxidative Stress and Apoptosis. Molecules. 2022;27.

22. Ververis A, Kyriakou S, Paraskeva H, Panayiotidis MI, Plioukas M, Christodoulou K. Chemical Characterization and Assessment of the Neuroprotective Potential of Euphrasia officinalis. Int J Mol Sci. 2024;25:1–17.

23. El-Sherbiny GM, Alluqmani AJ, Elsehemy IA, Kalaba MH. Antibacterial, antioxidant, cytotoxicity, and phytochemical screening of Moringa oleifera leaves. Sci Rep. 2024;14:1–17.

24. Petreska J, Stefova M, Ferreres F, Moreno DA, Tomás-Barberán FA, Stefkov G, et al. Dietary burden of phenolics per serving of “mountain tea”(Sideritis) from macedonia and correlation to antioxidant activity. Nat Prod Commun. 2011;6:1305–14.

25. Alsaud N, Farid M. Insight into the influence of grinding on the extraction efficiency of selected bioactive compounds from various plant leaves. Appl Sci. 2020;10.
